# Occupational airborne exposure, specific sensitization and the atopic status: evidence of a complex interrelationship

**DOI:** 10.1186/1745-6673-8-2

**Published:** 2013-02-13

**Authors:** Xaver Baur, Liubov Barbinova

**Affiliations:** 1 Institute for Occupational Medicine, Charité Universitätsmedizin Berlin, Thielallee 69, Berlin D-14195, Germany; 2 European Society for Occupational and Environmental Medicine, Berlin, Germany; 3 Institute for Occupational and Maritime Medicine, Universitätsklinikum Hamburg-Eppendorf, Hamburg, Germany

**Keywords:** Occupational exposure, Allergic asthma, Latex sensitization, Isocyanate asthma, Environmental sensitization, Atopy status

## Abstract

**Background:**

We have investigated the relationship between atopic status and long-term occupational exposure to latex proteins or methyl diethyl diisocyanate (MDI) as high and low molecular weight asthma-inducing agents, respectively.

**Methods:**

This study is based on retrospective analyses of two groups of symptomatic outpatients: 184 healthcare workers with latex exposure and 156 workers with isocyanate (MDI) exposure. We analysed atopic and non-atopic subgroups according to exposure duration and the frequencies of specific sensitization.

**Results:**

45% of the healthcare subgroup specifically sensitized to latex were atopic, whereas in the non-sensitized healthcare subgroup only 26% were atopic. On the other hand, subjects specifically sensitized to MDI were rarely atopic (only 15%), whereas in the subgroup non-sensitized to MDI atopy was present in 38%. After prolonged durations of exposure, the proportion of atopics was further elevated in most healthcare subgroups but it decreased in the MDI-exposed subjects.

**Conclusions:**

We hypothesize that latex proteins as sensitizing agents might promote the development of atopy, whereas exposure to the low molecular weight MDI might inhibit the atopic status.

## Introduction

Atopy, the tendency to induce IgE responses and IgE-mediated allergies to “trivial” concentrations of environmental allergens, has been defined in different ways – either from a pre-existing history of atopic disorders, increased total IgE levels or immediate-type sensitization to ubiquitous allergens, with the last definition currently favoured. Nowadays, 20 to 35% of the population are affected by atopy associated with allergic diseases of the skin, airways, conjunctiva, gastrointestinal tract and systemic reactions, making atopy an important healthcare problem [1-3].

The relationship between atopy and asthma is complex and is not fully understood, with genetic associations as well as discrepancies being described. About 60% of asthmatics are atopic and about one third of atopics exhibit bronchial hyperresponsiveness, a typical feature of asthma [4,5].

## The atopic status in occupational asthma

### Atopy identified as a risk factor

Cullinan et al. [6] examined 342 employees working in animal laboratories and found a strong positive association between pre-existing sensitization to environmental protein allergens and the development of newly occurring work-related asthmatic symptoms. Occupational asthma among bakers and millers has been well investigated and all authors have found a positive correlation between atopy and the development of asthma. Walusiak et al. [7] interpreted atopy as a confounder in the sensitization to flour allergens and Skjold et al. [8] identified atopy as a risk factor for work-related asthma symptoms and rhinitis. Baur et al. [9] determined that atopic bakers demonstrated asthma symptoms significantly more frequently than non-atopic ones. Similarly, the study of seafood handlers and caterers showed a positive association between atopy and occupational skin diseases induced by crustacea [10].

More controversial results have been obtained in investigations of low molecular weight, mainly irritant, isocyanates. Tarlo et al. [11] and Pronk [12] reported a negative association between atopy and asthma development, whereas others found no evidence of an association [13] or a positive one [14].

### Atopy as a response variable of occupational (environmental) airborne exposure

Several epidemiological studies indicate that the level, partially also the duration, of airborne exposure to specific substances can influence the atopic status. For example, endotoxin exposure in early childhood leads to a shift in the Th1 response and thus protects against atopic diseases [15,16]. Recently, similar findings have been described in adults [17-19]. There are indications that other non-allergenic agents also have an inhibitory effect on the atopic status. A negative correlation for instance between personal or parental smoking and atopy was described [20-22]. Sunyer et al. (1997) found a positive association between smoking and bronchial hyperresponsiveness in non-atopics and a lack of the association in atopics. They suggested that this may be due to the biological (immunological) antagonism between atopy and smoking [23].

Malo and co-workers (2000–2003) systematically investigated the effect of occupational substances on sensitization to specific occupational allergens as well as common allergens. They observed an association between new sensitizations to laboratory animals and to other animal allergens during the training period of apprentices in animal health technology. Gautrin et al. [24] found that veterinary, baker and dental technician apprentices who handled sensitizing substances had a high atopy incidence.

The reduced atopy frequencies in asthmatics sensitized to cleaning agents or other irritants [25] and an inverse relationship with the duration of occupational exposure to acid anhydrides [26] suggest that at least some irritant agents might suppress the development of atopy.

### An ambivalent role of atopy in occupational settings

The above-mentioned findings led us to formulate the hypothesis that atopy behaves in an ambivalent way and is not only predestined to act as risk factor in occupational asthma but can be promoted or inhibited by other airborne exposure in the working environment. The latter state can be indicated by changes in either sensitization to environmental allergens, the total IgE serum level or associated parameters. This issue has, however, been scarcely addressed. Inevitably, occupational and environmental allergies frequently overlap because of the structural similarity of the causative allergens, such as flour and grass pollen allergens, latex and several fruit allergens, laboratory animals and pets. There is evidence that the immune system responds to persistent allergen exposure by increasingly recognizing structurally related epitopes within allergen families [27]. On the other hand, airborne exposure to non-allergenic irritative substances, such as smoke, can - as already mentioned - obviously suppress the development of allergy.

**Figure 1:**
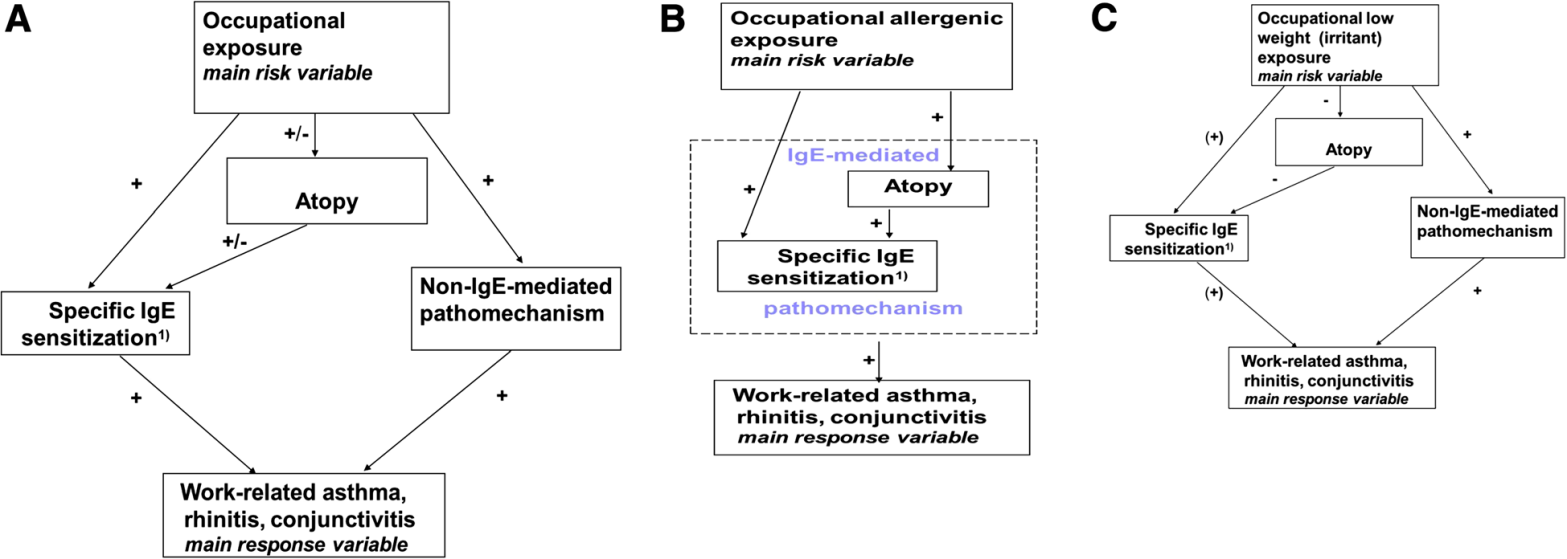
**A. Pathomechanic scheme of work-related asthma.** Specific sensitization and atopy represent response variables of the main risk variable ‘occupational exposure’ as well as explanatory variables of the definite response variable ‘work-related asthma’. For details see text. ^1)^ Broadening of sensitization to related allergens. **B**. Pathomechanic scheme of allergic asthma. Specific sensitization as a main mechanism of work-related asthma by exposure to high molecular latex allergens. The structural relations between factors are simplified in the case of a main pathomechanism. **C**. Pathomechanic scheme of isocyanate asthma. The main pathomechanism is unknown. The schema of Figure 1A cannot be principally simplified.

Given this pathomechanistic background, atopy can therefore be considered as a time-dependent variable which both affects the outcome, i.e. the main response variable occupational asthma and/or rhinitis (and other intermediate variables) and itself is affected by exposure (acting both as a risk factor and an additional response variable) (Figure 1A).

For this diversity, the term ambivalent seems appropriate.

To evaluate our hypothesis of an effect of occupational exposure on the atopic status we investigated the relationship between specific sensitization and atopy in two different exposed groups.

The idea for our analysis was that there is generally a positive association between exposure to occupational asthma-inducing agents (independent of their nature) and respective specific sensitizations and further that the relationship between atopy and occupational exposure depends on the nature of the occupational agent, i.e. that there is a positive association for allergenic substances and a negative one for irritants. This should lead to different findings in groups exposed to allergens, and irritants, respectively.

So, the aim of this retrospective study was to evaluate this hypothesis, namely to analyse the relationship between atopy and specific sensitization in two symptomatic groups exposed to sensitizing agents (latex allergens) or to a mainly irritant agent, methyl diethyl disocyanate (MDI). We related atopy parameters and specific IgE-sensitization to duration of exposure in an attempt to identify interrelationships.

## Methods

Our study was performed using retrospective analyses of 340 consecutively examined patients who attended our occupational medicine policlinic. All had complained about work-related respiratory symptoms, namely asthmatic symptoms (wheezing, chest tightness, dyspnea) and rhinitis, and belonged to two distinct occupational groups with an increased asthma risk: 184 healthcare workers in danger of developing IgE-mediated latex allergy, 156 subjects exposed to the isocyanate MDI and its oligomers, with mainly irritative effects on the airways, conjunctiva and skin.

The physician in charge completed a questionnaire for each patient, including a detailed occupational and clinical case history. For analyses, the data was stratified according to duration of occupational exposure into three groups (< 7 yrs, 7–15 yrs, >15 yrs) (Table 1).

**Table 1:** IgE-sensitization to the occupational agent in the latex and MDI groups according to exposure duration

	**Latex group (n = 184)**		**MDI group (n = 156)**	
	**Latex-sensitized**^**+ **^**n (%)**	**Latex-specific IgE mean (median); kU/L**	**MDI- sensitized**^**+ **^**n (%)**	**MDI-specific IgE mean (median); kU/L**
All (total groups)^#^	145/184 (78.8) ***	13.1 (5.7) *	20/156 (12.8)	6.3 (3.7)
Exposure = < 7 years	52/62 (83.9) ***	16.5 (6.3)	2/ 43 (4.6)	27.6 (27.6)
7 years < exposure = < 15 years	53/66 (80.3) ***	9.4 (5.7) *	6/39 (15.4)	1.6 (1.0)
Exposure > 15 years	26/40 (65) ***	11.9 (5.5) *	11/70 (15.7)	4.0 (4.3)

^**+**^ Sensitized = specific IgE antibodies detected.

^**#**^ In 16 subjects of the latex group (14 sensitized, 2 non-sensitized) and in 4 subjects of the MDI group (1 sensitized, 3 non-sensitized), estimations of exposure duration were not possible.

P values refer to comparisons between the respective latex and MDI groups: *** p <0.001; ** p <0.005; * p < 0.05.

Conclusive clinical diagnosis was always made in consultation with the senior physician on the basis of comprehensive clinical diagnostic parameters (including medical and occupational case history, results of lung function tests, NSBHR testing, allergological tests (see below), mostly also specific inhalative challenge tests or serial lung function tests [28].

Skin-prick tests for 17 common environmental allergens (*Dermatophagoides farinae, Dermatophagoides pteronyssinus*, dog hair/dog dander, horse hair/dander, cat hair/dander, feathers, *Alternaria alternata, Aspergillus fumigatus, Cladosporium herbarum*, mixed grass, tree pollen 1 (birch, hazel, alder), tree pollen 2 (ash, willow, poplar), tree pollen 3 (oak, beech), cow milk, egg white, rye flour, animal hair mixture) were performed. The skin-prick test was defined as positive when the corresponding allergen produced a wheal reaction ≥ 3 mm (in the absence of a reaction to saline). MDI-specific IgE-sensitization was assessed using MDI-human serum albumin by the CAP system (Phadia, Uppsala). The total IgE values were measured using the Uni-CAP system (Phadia, Uppsala). Subjects with a CAP value of > 0.35 kU/L were defined as latex-, and MDI-sensitized, respectively. We also examined a possible association between occupational exposure and specific IgE, skin-test reactions to environmental allergens and elevated total IgE blood levels (> 100 kU/L).

To analyse the association between atopy [29] and other parameters, we used two different categories of atopy severity on the basis of the skin-prick test results:

Atopy category 1 = at least one positive out of 17 environmental allergens in the skin-prick test.

Atopy category 2 = at least three positive out of 17 environmental allergens in the skin-prick test.

Where not otherwise stated, the term atopy (or atopic status) is used for skin-prick test responses to at least one allergen, corresponding to atopy category 1.

Finally, we compared atopic parameters in subgroups with various durations of exposure and the percentage of specifically sensitized subjects in these occupational groups.

Univariate comparisons and correlations were performed using appropriate parametric and nonparametric tests. Fisher’s exact test (or Mantel-Haenszel test) was used to estimate the differences. Data are expressed as mean ± SEM and p < 0.05 was regarded as significant. SAS 8.1 software was used for statistical calculations.

## Results

### Symptoms, atopy status and conclusive clinical asthma diagnosis

In the latex group, 80 subjects (43%) were diagnosed with occupational allergic asthma and 86 (47%) with occupational allergic rhinitis. In the isocyanate group, 66 subjects (42%) suffered from occupational asthma and only four subjects exclusively from occupational rhinitis. Work-related dyspnea was strongly associated with the conclusive diagnosis of asthma in the latex group OR = 21.5 (p < 0.0001) as well as in the isocyanate group OR = 17.6 (p < 0.0001).

In the latex group, there were no significant differences in frequencies of specific IgE sensitization or in atopy for the analysed subgroups, i.e. those with and without the diagnosis of occupational asthma. In the isocyanate group, all IgE-sensitized subjects were diagnosed with occupational asthma; the proportion of atopic isocyanate workers was significantly higher in the subgroup without occupational asthma than the subgroup with occupational asthma (41% vs. 26%; p < 0.05).

### Frequencies of specific sensitization in the latex and MDI groups

79% (145/184) of the latex group and 13% (20/156) of the MDI group individuals exhibited specific IgE antibodies against their occupational allergen (Table 1).

The frequency of specific IgE sensitization is high, but is slightly lower (ns) for the sensitized latex group with an exposure duration > 15 yrs, whereas IgE sensitization in the MDI subgroups is much lower with significantly increased frequency after longer exposure durations (exposed for > 7 yrs) (p < 0.05) (Table 1).

### Atopy frequencies in the latex and MDI groups with different exposure duration

When comparing the groups (all; Table 2), only a slight (ns) difference in the proportions of atopy is observed, i.e. 41% in the total latex group vs. 35% in the total MDI group for atopy category 1, and 25% vs. 19% for atopy category 2. The assessment of these atopic parameters as a function of exposure duration revealed that the frequency of environmental sensitization increased in the latex group after more than seven years (ns). On the other hand, a significantly negative correlation between the proportion of atopics and the duration of exposure (r_Spearman_ = −0.2; p < 0.05) is demonstrated in the MDI group.

**Table 2:** Distribution of sensitization to common allergens in the latex and MDI subgroups according to duration of exposure

	**Latex-exposed subjects (n = 184)**			**MDI-exposed subjects (n = 156)**			**P values**
	**All n = 184**	**Latex sensitized**^**+ **^**n = 145 (78.8%)**	**Latex non-sensitized n = 39 (21.2%)**	**All n = 156**	**MDI sensitized**^**+ **^**n = 20 (12.8%)**	**MDI non-sensitized n = 136 (87.2%)**	
Age (mean); years	32.5	31.6	35.8	41.4	41.5	41.4	
M/W	18 /166	15 /130	3 /36	139 /17	15 /5	124 /12	
Exposure duration; mean (median) years	11.4 (9)	10.8 (9)	13.6 (11)	16.5 (14)	16.8 (18)	16.5 (13)	
Exposure groups; n (%)	168 (100)	131 (78)	37 (22)	152 (100)	19 (12.5))	133 (87.5)	
Exposure = < 7 years	62 (36.9)	52/62 (83.9)	10/62 (16.1)	43 (28.3)	2/ 43 (4.6)	41/ 43 (95.4)	
7 years < exposure = < 15 years	66 (39.3)	53/66 (80.3)	13/66 (19.7)	39 (25.6)	6/39 (15.4)	33/39 (84.6)	
Exposure > 15 years	40 (23.8)	26/40 (65)	14/40 (35)	70 (46.1)	11/70 (15.7)	59/70 (84.3)	
Atopy category 1; n (%)	75/184 (40.7)	65/145 (44.8)	10 /39 (25.6)	54/156 (34.6)	3/20 (15)	51/136 (37.5)	**
Exposure = < 7 years	23/62 (37.1)	22/52 (42.3)	1/10 (10)	20/43(46.5)	1/2	20/41 (48.8)	
7 years < exposure = < 15 years	29/66 (43.9)	25/53 (47.2)	4/13 (30.7)	15/39 (38.5)	1 /6	14/33 (42.4)	
exposure > 15 years	17/40 (42.5)	12/26 (46.2)	5/14 (35.7)	17/70 (24.2)	1/ 11	15/59 (25.4)	
Atopy category 2; n (%)	46/184 (25.0)	43/145 (29.7)	3/39 (7.7)	29/156 (18.6)	0	29/136 (21.3)	***
exposure = < 7 years	12/62 (19.3)	12/52 (23.1)	0/10 (0)	10/43 (23.2)	-	10/41 (24.4)	
7 years < exposure = < 15 years	20/66 (30.3)	19/53 (35.8)	1/13 (7.7)	9/39 (23.1)	-	9/33 (27.2)	
Exposure > 15 years	10/40 (25)	8/26 (30.8)	2/14 (14.3)	7/70 (10)	-	8/59 (13.6)	
Total IgE mean (median), kU/L ^#^	286.3 (105.7)	334.3 (168)	73.4 (29.3)	171.6 (74)	135.0 (77.3)	176.5 (74)	
Exposure = < 7 years	329.7 (130.5)	373.5 (169)	101.7(23.2)	187.3 (95.7)	246.6 (246.6)	184.1 (95.7)	
7 years < exposure = < 15 years	264.3 (116)	309.9 (165.5)	81.7 (49.9)	205.1 (69)	97.7 (64.5)	223.4 (70.3)	
Exposure > 15 years	231.6 (91.4)	329.3 (151.5)	50.2 (33)	118.0 (64.5)	131.1 (88.4)	115.5 (60)	
Total IgE elevated; n (%)	94/182 (51.6)	89/143 (62.2)	5/39 (12.8)	60/144 (41.8)	9/19 (47.4)	51/125 (40.8)	**
Exposure = < 7 years	33/62 (53.2)	32/52 (61.5)	1/10 (10)	19/40 (47.5)	1/2 (50)	18/38 (47.4)	
7 years < exposure = < 15 years	36/65 (55.4)	34/52 (65.4)	2/13	14/34 (41.2)	2/5 (40)	12/29 (41.4)	
Exposure > 15 years	18/40 (45)	16/26 (61.5)	2/14	25/68 (36.8)	5/11 (45.4)	20/57 (35.1)	

^**+**^ Sensitized = specific IgE antibodies detected.

^**#**^ In 2 subjects of the latex group (both sensitized) and in 12 subjects of the MDI group (1 sensitized, 11 non-sensitized the total IgE level could not be measured.

P values are presented for exposure differences (latex vs. MDI) in odds ratios relating specific sensitization and atopy (Mantel-Haenszel statistics).

*** p < 0.001; ** p < 0.005; * p < 0.05.

### Atopy frequencies in the sensitized and non-sensitized latex and MDI subgroups

**Figure 2:**
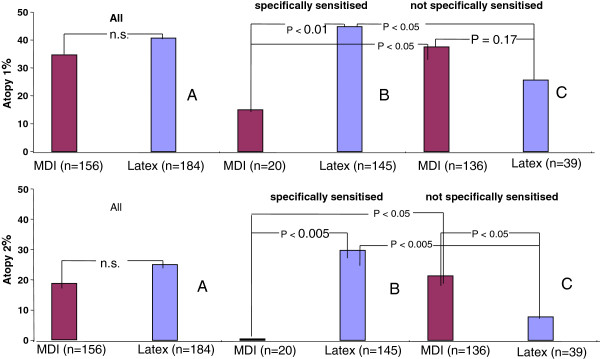
**I: Percentage of atopy grade 1 in the latex and MDI subgroups: (A – all; B – specifically sensitized; C – not specifically sensitized).** Healthcare workers sensitized to latex were significantly more frequently atopic than non-sensitized healthcare workers. The converse relationship was found for MDI workers. **II**: Percentage of atopy grade 2 in the latex and MDI subgroups. Differences between occupational subgroups were more pronounced for atopy grade 2, e.g. none of the 20 MDI-sensitized workers and only 8% of the non-sensitized latex workers were designated atopy grade 2.

Differences are found after redistributing the latex and MDI-exposed employees, according to their occu-pationally specific IgE findings, into those specifically sensitized (IgE > 0.35 kU/L) or non-sensitized to their occupational allergen (IgE ≤ 0.35 kU/L). The proportion of atopics in the latex-sensitized subgroup is significantly higher than for the non-sensitized subgroup (45% vs. 26%; p < 0.05 for atopy category 1; 30% vs. 8%; p < 0.005 for atopy category 2; Figure 2, Table 2).

In contrast, the proportion of atopics is significantly lower in the MDI subgroup with IgE antibodies to MDI-HSA conjugates than in the subgroup of non-sensitized MDI workers (15% vs. 38%, p < 0.05; Figure 2, Table 2). This difference is more evident in the higher atopy category 2 (0 vs. 21%; p < 0.05).

Consequently, the association between specific and environmental sensitization is positive in the latex group and negative in the isocyanate group (atopy 1: OR = 2.4, p < 0.05 vs. OR = 0.3, p = 0.08; atopy 2: OR = 5.1, p < 0.005 vs. cohort risk = 0.8, p < 0.05).

### Atopy frequencies in the sensitized and non-sensitized latex and MDI subgroups according to duration of exposure

Whereas the frequency of latex-specific sensitization is slightly reduced in those with a longer duration of exposure, the frequency of atopy is slightly elevated. Conversely, MDI sensitization in the MDI-exposed subjects is significantly increased in the subgroups with longer durations of exposure, but the number of atopic individuals is significantly less. This trend is opposite to that observed for the latex group and is pronounced both in the specifically sensitized as well as in the non-sensitized MDI subgroup (Table 2).

### Frequencies of increased total IgE levels in the sensitized and non-sensitized latex and MDI subgroups

**Figure 3:**
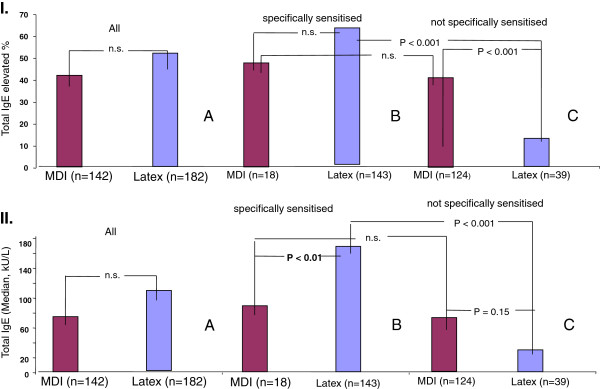
**Total IgE levels in the latex and MDI subgroups. I**: Percentage of subjects with elevated total IgE serum levels ( > 100 kU/L) in the latex and MDI subgroups. The relationship between the proportions of subjects with elevated total IgE levels in the sensitized and non-sensitized groups was similar to their respective atopy relationships in Figure 2, but less pronounced. **II**: Absolute total IgE serum level (medians) in latex and MDI subgroups. The relationship between absolute total IgE values in sensitized and non-sensitized subgroups was similar to the relationship between percentages of atopics in Figure 3.I.

The total IgE levels are significantly higher among latex-exposed subjects than in the MDI-exposed subjects (286 vs. 172 (kU/L); p < 0.005). The proportion of patients with an increased total IgE level (> 100 kU/L) is significantly higher among latex-sensitized employees than in non-sensitized latex-exposed subjects, whereas there are no significant differences in the equivalent MDI subgroups. Accordingly, the difference in the proportions of increased total IgE levels in subgroups (occupationally specifically sensitized vs. non-sensitized) is significant (p < 0.001) between the latex-exposed group (62% vs. 13%) and the MDI-exposed group (47% vs. 41%), (Figure 3.I). The same applies to the median of the total IgE level (Figure 3.II).

### Frequencies of increased total IgE level in the sensitized and non-sensitized latex and MDI subgroups according to duration of exposure

Latex-exposed employees placed into subgroups ac-cording to their duration of exposure did not reveal significant differences in total IgE concentrations, which is mainly in agreement with the skin-prick test results (Table 2). A significant trend towards decreased total IgE concentrations for increasing exposure periods (p < 0.001) was observed for the group of MDI-exposed subjects. This result is also in accordance with the findings for the skin-prick tests with ubiquitous allergens (Table 2).

## Discussion

We found that more than three quarters of the asthmatic healthcare group but only one eighth of the asthmatic MDI workers show specific sensitization. This corresponds to other studies indicating that latex represents a potent inhalative allergen [30] whereas the great majority of affected isocyanate workers exhibit no overt immune response [31,32]. Also the time trends in these groups are different; the frequency of latex sensitization is slightly reduced in those with longer periods of exposure whereas MDI sensitization significantly increased in the MDI-exposed subgroup with longer durations of exposure.

In comparison with the general population, the proportion of atopic individuals is increased in the latex group and lower in the isocyanate group. These trends become reinforced with longer exposure periods to latex, with an even higher frequency of atopy after seven years or more; on the other hand there is a further, significantly reduced frequency after prolonged MDI exposure. These associations are particularly evident in the higher atopy category 2.

An additional remarkable result of our study is that there were more pronounced significant differences in the proportions of atopics between the subgroups that were specifically sensitized and non-sensitized, respectively, to their occupational agents. Nearly half of the latex-sensitized subjects (45%) are sensitized to environmental allergens. Among MDI-sensitized employees, only every seventh (15%) is sensitized to environmental allergens (p < 0.01). This discordant effect is more evident in the subgroups with longer periods of exposure.

Given that occupational exposure represents an initiative cause that introduces the work-related sensitization, we can assume that the positive association between specific sensitization and atopy in the latex group, and the negative association in the MDI group is due to diverse biological effects and is induced by pathomechanic effects of the causative agents.

### Interpretation of the relationship between specific sensitization and atopy: possible pathomechanisms

To fully appreciate the significance of our findings, we must take into account that our study was of a cross-sectional design with a collection of patients over an extended time span which might have been associated with some selection bias. Primarily, conditioning on symptoms and/or diagnosis (case-only study) may introduce a collider bias [33], i.e. the spurious associations that cannot be accorded to real biological associations between factors. Secondarily, a healthy worker effect, i.e. exclusion of subjects from work and the study on the basis of symptoms and abnormal findings, might have taken place. Hence, by interpretation of statistical associations in groups with symptoms we have to estimate how the study design and a possible selection bias affected the outcome. Nevertheless, we have to keep in mind that all these processes (the true biological associations as well as different forms of distortion due to biases) have their background in real pathomechanisms of the disease. Accordingly, the provision of insight into the pathomechanisms of the disorder is the ultimate arbiter of the interpretation of study results.

Each interpretation of statistical associations as real biological interactions implies that the considered factors are involved in the same pathomechanism, and the biological background of interaction is known (or it is at least hypothesized); that there is a natural basis for the interpretation) [34]. However, this is a necessary, not a sufficient condition. Such an interpretation excludes other factors or considers them as not important. Evidently, if and only if one of the considered risk factors is a major risk factor of disease, other factors may be regarded as negligible. Also, the knowledge about the main factor of a disease plays a decisive role for the adequate interpretation of statistical associations between factors.

Theoretically, two principally different causal schemes of pathogenesis have become possible:

1) the disease originates from a main pathomechanism; this implies that the major pathomechanistic factor is known (as a rule, the name of the pathomechanism is derived from this factor).

2) the major pathomechanism is unknown or there is no major pathomechanism, eventually the disease arises through two (or more) different pathomechanisms; the knowledge about some minor pathomechanisms may be available.

We see the essential difference between these two schemes. The first case is much easier to analyse than the second. In such a situation we may, along with the biological background, interpret the statistical associations between the main factor and other risk factors, involved in the major pathomechanism, as true biological interactions.

There is evidence that this is exactly the case for latex (79% of the latex-exposed group were IgE-responders to latex).

In the case of a major pathomechanism of asthma the scheme in Figure 1A is reduced to the simpler scheme in Figure 1B. For simplicity of analysis, the main mechanism can be considered as a sole mechanism and the other pathomechanisms can be neglected.

#### Possible pathomechanistic background for effects of occupational exposure to allergic agents on specific sensitization and atopy

The afore mentioned findings indicate that a positive association between latex sensitization and atopy can be interpreted as being due to biological interactions between structurally related protein epitopes of the occupational latex and environmental allergens which develop during the exposure period.

We have to examine how the case-only design of our study may have affected the results, i.e. to which extent the obtained ORs are unbiased estimates for the joint effect of factors in a standard case–control study. The equivalence of case-only and case–control studies has been thoroughly investigated in the context of gene-environment interaction studies. Correspondingly, the necessary assumptions for the equivalence have been defined within the framework of these studies, namely, i) the disease/its symptoms is rare; ii) the considering factors occur independently in the source/control population [35,36]. The first assumption deals with general constructive conditions for odds-ratios models (it provides that the distribution of participants with each combination of factors in the control study group is the same as in the source group) and can be generally accepted. This condition is fulfilled in both our study groups: even in health care workers highly exposed to latex, the prevalence of occupational asthma was not higher than 2-10% [30], or 1–4% [37,38]; for isocyanates annual occupational asthma incidence rates have been evaluated as < 1%, with a prevalence between 5-10% [39-41]. However, the second assumption cannot be unscrupulous transferred to occupational settings. Whereas the factors considered in gene-environment studies remain constant, in occupational settings may be present the factors which are changed due to exposure. Specific sensitization is one exact extreme case: without occupational exposure no corresponding specific sensitization exists, the occupational exposure is the initiative cause of specific sensitization. The relationship between factors also arises during exposure. Obviously, the positive association between specific IgE-sensitization and atopy in symptomatic subjects is an integrated result from the interaction in both stages of the process leading to asthmatic symptoms via an IgE-sensitizing mechanism: in the stage of developing sensitization and in the stage of development of symptoms.

Hence, under the assumption that exposure and atopy occur independently in the source population we may consider the obtained association as an estimation for the joint effect of interaction between factors, namely, as a positive (synergistic) biological interaction (OR > 1) resulting in the development asthmatic symptoms via IgE-specific sensitization. We find this assumption reasonable since the likelihood of exposure is the same in atopics and non-atopics at the beginning of the work history (exposure).

If the main pathomechanism is known, it is also easy to assess the influence of the second form of bias - the healthy-worker effect - on constellations between factors in symptomatic subjects. In the case of latex asthma there is the simple corollary, i.e. specifically sensitized subjects are easily diagnosed with the use of allergological tests followed by quitting their jobs and being missed in groups with prolonged exposures. The decrease in latex sensitization and lower specific IgE values with longer periods of exposure may be due to these selective effects in the compilation of the subgroups.

**Figure 4:**
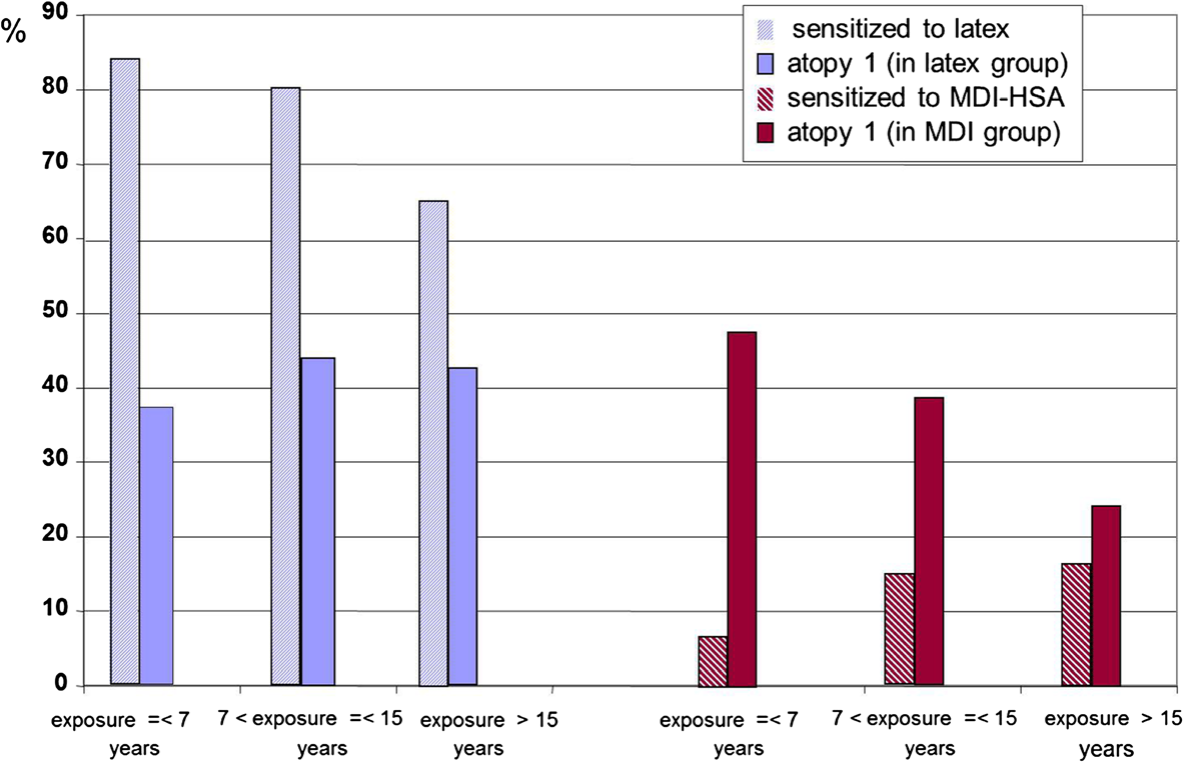
**Specific sensitization and atopy in the subgroups with different exposure durations.** The trend of relationship between atopy and specific sensitization across the subgroups is explicitly opposite for latex and isocyanate exposure.

Furthermore, taken into account the above consequences (we assumed that all bias processes have been reliably estimated), we can use the trend across the subgroups with different exposure durations for assessment of effects of exposure on atopy. On the basis of the negative selection of specifically sensitized subjects in the course of exposure, a parallel negative selective process of atopics can be expected. But the observed trend by atopics is increased (Figure 4); and it is strongly pronounced in the non-sensitized latex group (Table 2). We can interpret this trend as evidence that latex exposure may promote the atopy status both in the specifically sensitized as well as in the non-sensitized subgroup (see Figure 1B).

Results of Gautrin et al. [42], Nguyen et al. [43] and Skjold et al. [8] confirmed the hypothesis of biological interactions between atopy and exposure to allergenic substances. These authors observed an increase in the atopy incidence over a period of about three years among apprentices exposed to different allergens, even in the absence of specific sensitization, with significant increases in sensitization in the molds and pets group.

Taken all together, these studies as well as our own findings suggest that exposure to sensitizing protein allergens may promote the development of atopy.

#### Possible pathomechanistic background for effects of occupational exposure to mainly irritative agents on specific sensitization

As opposed to high molecular weight compounds such as latex allergens, specific IgE sensitization is not a major pathomechanism in isocyanate asthma (it is only detected in 10-30% of affected isocyanate workers [32,44]); the non-IgE mediated form accounts for the majority of isocyanate asthma (87% in our study). So as opposed to latex, isocyanate asthma does not imply a single pathomechanism. There are at least two forms: i) an IgE-depended and ii) an IgE-independent non-immunological irritant form (isocyanates are potent irritants evident especially at high isocyanate concentrations) [31,45]. Accordingly, the schema of Figure 1A cannot be simplified in case of isocyanate asthma (Figure 1C).

Atopy as a risk factor can be involved in different ways (in various paths) in the genesis of non-immunological isocyanate asthma; and the constellations in this subgroup may essentially affect (by selection processes) the relations between the factors in the small IgE-mediated subgroup. It is exactly in this situation that a collider bias can be expected. According to causal models, a collider bias reflects a general phenomenon known as Berkson’s paradox, when conditioning on a common consequence of two independent causes renders these causes statistical dependent (for details see J. Pearl [46]).

Simply, when the factors belong to independent pathomechanistic ways then the negative statistical associations are due to composition of those different pathways and do not reflect true biological relations between risk factors. Also in a situation, when one factor is involved in some pathomechanisms (perhaps at different times), the multiple connections may cause a selection bias inducing spurious relationships with other factors. This can be the reason for distinct assessment of relations between factors in isocyanate asthma when comparing the results from various studies. Therefore, for isocyanate-exposed subjects we cannot directly interpret the constellations between specific sensitization and atopy as biological such as in the case of the latex group.

The time trend of atopy represents an example of the possibility of such a complex relationship. We found a clear negative trend over time for atopy in the whole MDI group as well in the non-sensitized MDI subgroup (r_Spearman_ = − 0.2; p < 0.05); thereby in the shorter exposure set (< 7 years) atopics are found twice as frequently (49%) than in the group of prolonged exposure (25%). Because of the heterogenous pathomechanistic process we cannot assess whether it is a suppressing effect of exposure or the constellation of different time-dependent forms (e.g. atopics may develop symptoms earlier, so being reduced in subgroups with longer exposure). Accordingly, the process of specific sensitization which occurs mostly after long exposure periods may be affected. Interestingly, the only study in which a positive association between atopy and isocyanate asthma was reported (Meredith et al., [14]) had used a definition of atopy based on a pre-employment history of hay fever, eczema and/or asthma. However, the authors found no association between duration of exposure and atopy.

The decrease of symptomatic atopic isocyanate workers over time is remarkable and could be due to the following reasons: i) the negative selection of the atopics (e.g. on the basis of an earlier development of non-IgE-mediated isocyanate asthma in atopics), ii) a protective effect of longer isocyanate exposure upon the development of atopy.

The study of Hur et al. [47] showed a similar constellations than our: atopics were less frequently found (15% vs. 31%) among those with work-related respiratory symptoms and all subjects with diagnosed isocyanate asthma or isocyanate-induced eosinophilic bronchitis (n = 7; 4 of those with IgG) were non-atopic; however, the authors did not discuss these observations.

The presence of different pathomechanisms in isocyanate asthma makes it impossible to assess the selection trend over time in our study as well as in others with a case-only design.

Nevertheless, if we take into account the studies with exposed workers (where the negative selection occurs less likely than in asthmatic subjects), we find agreement with our findings, too. Namely, a drastic reduction in atopy frequency was recently reported in a cross-sectional study of spray painters with a relatively high exposure to isocyanates [48] as well as in other isocyanate workers [11]. Recently, an immunological mechanism mediated by IgG antibodies has been observed. However, there was no association between symptoms and specific IgG finding, obviously the latter simply serve as a marker of exposure and not as a marker of disease [49]. There is even speculation about a possible protective effect of IgG or IgG4 on the incidence of work-related symptoms [39].

Taken together, there is no available data for isocyanate asthma patients or those exposed to isocyanate that resolves this dilemma of decrease of atopics under isocyanate exposure.

There is evidence also for involvement of genetic factors in the pathomechanism of isocyanate asthma. Some variants of polymorphisms within genes of the gluthathione S-transferase (GST) superfamily (e.g. GSTP1Val/Val) have been associated with susceptibility to isocyanate-induced asthma [41].

Further, the study of Piirila et al. [50] investigating the role of N-acetyltransferase (NAT) genotypes found that the slow acetylator genotype (NAT1) conferred a 2.5-fold increase in risk for isocyanate asthma. Recently, Bernstein [51] described a significant association between IL4RA and isocyanate asthma but only for subjects exposed to HDI, and not to MDI and TDI. Nevertheless, these genetic determinations can be realised by various pathomechanistic pathways. This makes the associations ambiguous and dependent on the contributions of other factors (e.g. downstream/epistatic genes) in the pathways. Additionally, the variety of environmental and occupational factors can interact with the genetic determinants and affect the development of disease [52]. Notably, Mapp al. reported a decreased risk of isocyanate OA in the GSTP1Val/Val genotype [53].

Despite the differences and complexity, it is notable that loci showing strong association with allergen-induced asthma (IL13, FCER1A) were not found to be associated with isocyanate asthma [52], which may support our hypothesis of two quite different (partially genetically determined), atopic and non-atopic immunological responses (see above).

In summary, the genes responsible for the primary and/or secondary regulation of pathomechanistic pathways of isocyanate asthma might be common and it is their expression or their manifestation that could be affected by multiple environmental as well as occupational factors. It can be assumed that prolonged exposure to isocyanates might increase the expression of genes associated with disease susceptibility. Further, we speculate that these processes are negatively conjugated with the processes of development of atopy.

## Conclusions

We hypothesize that the significant difference between the two investigated occupational asthma groups, latex- and MDI-exposed subjects, arises from the work-related exposure and not from selection effects. This phenomenon could have a genetic and/or epigenetic basis, involving differences in recognition, processing or in specific immunological responses to isocyanate conjugates as opposed to foreign allergenic proteins, such as latex. The antigenic structures of MDI-HSA conjugates (Baur et al. [54]) are quite different from latex and environmental protein allergens. IgE-mediated sensitization to the latter is defined as atopy and atopic patients may easily develop sensitization to similarly structured latex proteins but not to conjugates of low molecular weight chemicals with human serum protein. The immune system seems preconfigured in a particular way (atopy or non-atopy) and in the case of atopy can easily respond to similar protein epitopes. However, longitudinal environmental exposure might lead to a switch from the atopic to the non-atopic status or *vice versa* depending on the nature of the environmental agent.

The results in our latex and MDI groups with discrepant frequencies and development of the atopic status as well as the increase of specific sensitization to MDI (which does not belong to allergens recognized by atopics) after prolonged exposure (see Table 2) are in agreement with this preposition.

The classic scheme of regarding atopy only as a risk factor should be reconsidered. Rather, atopy may exhibit an ambivalent role as a risk factor as well as being an intermediate additional response variable (explanatory variable) (Figure 1A).

Interestingly, there is evidence that not only exposure to isocyanates but also exposures to other irritants or low molecular weight substances [22], such as bleach [55] or endotoxins [4,19,31,56], are associated with a reduction in the frequency of atopic sensitization.

However, whether exposure to low molecular weight chemicals, such as isocyanates, may have a direct inhibitory effect on the atopic status cannot definitively be resolved from our data.

More investigations of well-defined exposures in occupational and environmental settings are required to elucidate precisely the environmental influence on specific sensitization and asthma development in relation to the atopic status.

## Competing interest

The authors declare that they have no conflict of interest.

## Authors’ contributions

The manuscript has been read and approved by both authors. Both authors participated substantially in analysis of data, discussion and development of concept.
